# Cyclooxygenase Inhibition Alters Proliferative, Migratory, and Invasive Properties of Human Glioblastoma Cells In Vitro

**DOI:** 10.3390/ijms22094297

**Published:** 2021-04-21

**Authors:** Matthew Thomas Ferreira, Juliano Andreoli Miyake, Renata Nascimento Gomes, Fábio Feitoza, Pollyana Bulgarelli Stevannato, Andrew Silva da Cunha, Fernanda de Oliveira Serachi, Alexandros Theodoros Panagopoulos, Alison Colquhoun

**Affiliations:** 1 Department of Cell and Developmental Biology, Biomedical Sciences Institute, University of São Paulo, 05508-000 São Paulo, Brazil; matthew.t.ferreira@gmail.com (M.T.F.); renataalex@usp.br (R.N.G.); medicyfabio@hotmail.com (F.F.); pollystevanatto@hotmail.com (P.B.S.); andrew_scunha@hotmail.com (A.S.d.C.); fernandaserachi@gmail.com (F.d.O.S.); 2 Department of Morphological Sciences, Biological Sciences Centre, Federal University of Santa Catarina, Campus Trindade, Mailbox 476, 88040-900 Florianópolis, Brazil; juliano.miyake@ufsc.br; 3 Department of Neurosurgery, Santa Casa Hospital, 01221-020 São Paulo, Brazil; allexandros@bol.com.br

**Keywords:** cyclooxygenase, prostaglandin, glioblastoma, matrix metalloproteinase, migration

## Abstract

Prostaglandin E_2_ (PGE_2_) is known to increase glioblastoma (GBM) cell proliferation and migration while cyclooxygenase (COX) inhibition decreases proliferation and migration. The present study investigated the effects of COX inhibitors and PGE_2_ receptor antagonists on GBM cell biology. Cells were grown with inhibitors and dose response, viable cell counting, flow cytometry, cell migration, gene expression, Western blotting, and gelatin zymography studies were performed. The stimulatory effects of PGE_2_ and the inhibitory effects of ibuprofen (IBP) were confirmed in GBM cells. The EP2 and EP4 receptors were identified as important mediators of the actions of PGE_2_ in GBM cells. The concomitant inhibition of EP2 and EP4 caused a significant decrease in cell migration which was not reverted by exogenous PGE_2_. In T98G cells exogenous PGE_2_ increased latent MMP2 gelatinolytic activity. The inhibition of COX1 or COX2 caused significant alterations in MMP2 expression and gelatinolytic activity in GBM cells. These findings provide further evidence for the importance of PGE_2_ signalling through the EP2 and the EP4 receptor in the control of GBM cell biology. They also support the hypothesis that a relationship exists between COX1 and MMP2 in GBM cells which merits further investigation as a novel therapeutic target for drug development.

## 1. Introduction

Primary brain tumors are estimated to be responsible for almost 2.5% of all cancer related deaths. Of all central nervous system (CNS) tumors, approximately 25.5% are gliomas and of all malignant CNS tumors approximately 80.8% are gliomas. Astrocytic tumors including pilocytic astrocytoma, anaplastic astrocytoma, diffuse astrocytoma and glioblastomas (GBM) account for 76.4% of all gliomas. Of these astrocytic gliomas, GBMs account for 57.3%. Thus, the gliomas are by far the most common primary brain tumors, with a mortality rate higher than any other brain related disease except for brain related vascular problems, and patients with GBM have a dismal prognosis with a five-year overall survival rate of 6.8% [1]. Despite the efforts to treat GBM patients using the conventional therapies of surgical resection, radiation therapy and chemotherapy, the efficacy of these combined treatments is short-lived and the overall survival for most patients is between 12 and 15 months.

The inflammatory process is considered an important factor in tumor development and progression in general [2,3,4,5]. Certain eicosanoids, including prostaglandins and leukotrienes, are potent mediators of inflammatory processes [6,7,8]. Both the prostaglandin and leukotriene families of eicosanoids have been implicated in the formation and progression of tumors. The expression of the inducible form of cyclooxygenase, COX2, has been correlated with tumor progression and metastasis in several tumors including gastrointestinal, breast, prostate, ovarian and lung cancer [9,10]. Increased concentrations of prostaglandin E_2_ (PGE_2_) have been found in malignant lesions and the increased expression of COX2 and increased PGE_2_ are thought to be associated with a poor prognosis in most cases [3,4,5,11,12,13]. Both COX2 and microsomal prostaglandin E synthase 1 (mPGES1) are inducible enzymes typically upregulated in inflammatory situations and both are known to be overexpressed in several cancers [11,13,14,15]. PGE_2_ produced by the sequential activity of these two enzymes is a major contributor to tumor development and progression through its stimulation of cell survival, proliferation, migration, and invasion [10,11,16].

Recent studies from our laboratory have shown that PGE_2_ content was increased in GBM tumours in comparison with lower grade gliomas and the concentration of PGE_2_ was positively correlated with the content of arachidonic acid in GBM tissue. The study also showed a correlation between intratumoral PGE_2_ concentration and patient survival, where the higher the concentration of PGE_2_ the poorer was patient survival, with a median survival for high PGE_2_ of 3.5 months versus 11 months for low PGE_2_ [13].

Nonsteroidal anti-inflammatory drugs (NSAIDs), like ibuprofen (IBP) and aspirin, inhibit COX activity effectively. However, their long-term use has proven to be harmful to the liver and GI tract and they are not generally used as a long-term chemotherapeutic approach [17]. Despite this, the development of COX-targeting therapies with reduced side effects continues to be of interest due to the pro-tumorigenic and pro-angiogenic effects of lipid mediators produced by both COX1 and COX2 [18,19,20]. However, it is interesting that long-term aspirin use has been proposed to protect against glioma development in the human population [18]. A series of studies in our laboratory have shown that NSAIDs complexed with ruthenium in a novel paddlewheel structure have promising effects on GBM survival, proliferation and migration both in vitro and in vivo [21,22,23].

One hallmark of cancer partially responsible for the lack of successful therapies, is invasiveness [3]. GBM cells are capable of migrating and invading neighboring tissues early in the disease process, and there are several pathways responsible for transformed cells’ ability to relocate [24]. Matrix-metalloproteinases (MMPs) are zinc endopeptidases mainly responsible for the alteration of the extracellular matrix (ECM) and its biophysical properties [25,26]. MMP2 and MMP9 gelatinase isoforms are responsible for degrading type IV collagen in the ECM. Type IV collagen is a crucial component of the basement membrane, separating pericytes and astrocytes from the endothelium and is crucial for the proper function of the blood brain barrier (BBB) [20,27]. Type IV collagen expression is also often up regulated in glioma tissue [28]. In addition, MMP2 and MMP9 up-regulated expression has also been reported in primary GBM samples and in some cell lines. However, there are some controversies in the literature [29]. The therapeutic potential of MMP inhibition is very promising as reviewed by Levin et al. [30,31,32].

Despite the importance of the PGE_2_ pathway in GBM cells described previously [13,21,22,23], the relationship between MMP activity and COX pathways has not been thoroughly explored. In this study we examined the influence of COX1 and COX2 inhibition upon cell proliferation and migration, and MMP2, MMP9 and MMP14 expression in GBM cell lines in vitro.

## 2. Results

### 2.1. Cyclooxygenase Protein Expression in Glioblastoma Cells Lines and Surgically Resected GBM Tissue

**Figure 1:**
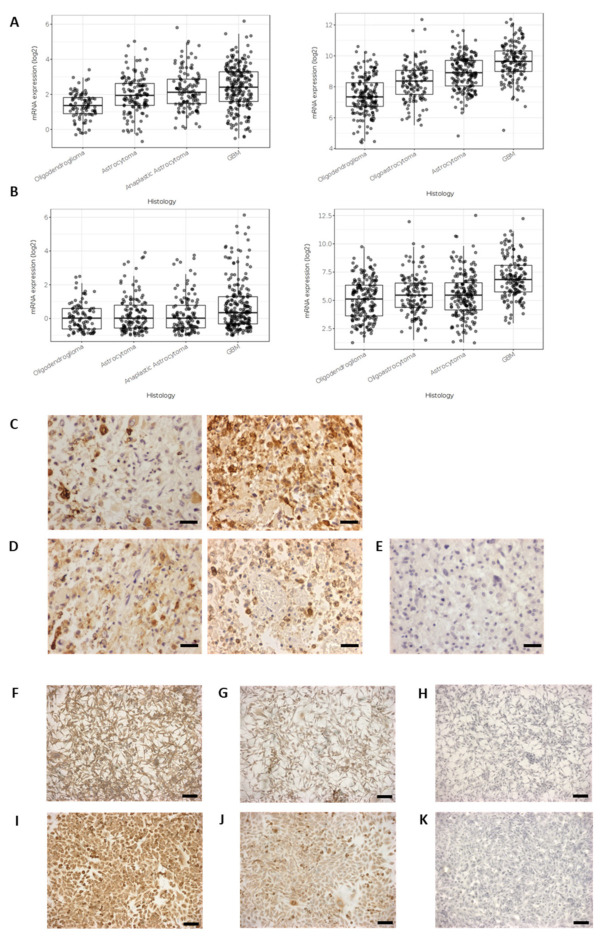
Cyclooxygenase expression in gliomas. (**A**) PTGS1 (COX1) mRNA expression in gliomas using Gliovis software for analysis, TCGA and CCGA datasets; (**B**) PTGS2 (COX2) mRNA expression in gliomas using Gliovis software for analysis, TCGA and CCGA datasets; (**C**) COX1 immunohistochemistry reaction in GBM tissue sections; (**D**) COX2 immunohistochemistry reaction in GBM tissue sections; (**E**) Negative control reaction in GBM tissue section; (**F**) COX1 immunocytochemistry in U87MG cells; (**G**) COX2 immunocytochemistry in U87MG cells; (**H**) Negative control, U87MG; (**I**) COX1 immunocytochemistry in U251MG cells; (**J**) COX2 immunocytochemistry in U251MG cells; (**K**) Negative control, U251MG. Scale bar in (**C**–**E**) = 30 µm; scale bar in (**F**–**K**) = 50 µm.

The mRNA expression of PTGS1 (COX1) and PTGS2 (COX2) is presented for differing grades of glioma using the Gliovis data portal for visualization and analysis of brain tumor expression datasets to analyze data from the TCGA and the CCGA [33]. The expression of both COX1 and COX2 are significantly increased in grade IV GBMs in comparison with lower grade gliomas (Figure 1A,B). The images presented in Figure 1C,D show the COX1 and COX2 immunohistochemistry reaction for surgically resected GBM tissue. The images presented in Figure 1F–K show the COX1 and COX2 immunocytochemistry reaction for two GBM cell lines, U87MG and U251MG, exemplifying the continued presence of COX1 and COX2 in established GBM cell lines as previously reported [22,34].

### 2.2. Effect of Exogenous PGE_2_ on GBM Cell Counts

**Figure 2:**
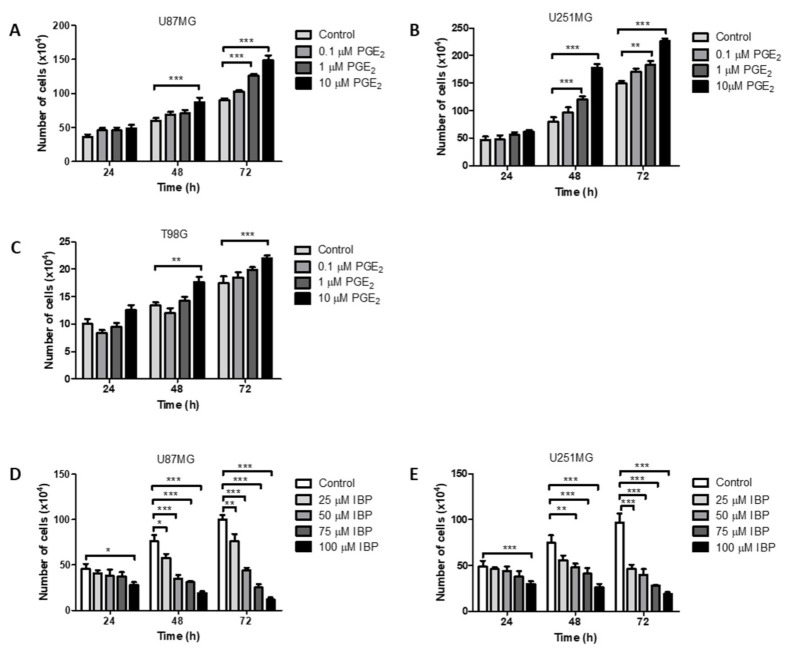
Opposing effects of PGE_2_ and COX1/2 inhibitor, IBP, on GBM cell counts. Graphs show the results after 24, 48 or 72 h of treatment with PGE_2_ or IBP. (**A**) PGE_2_ in U87MG cells; (**B**) PGE_2_ in U251MG cells; (**C**) PGE_2_ in T98G cells; (**D**) IBP in U87MG cells; (**E**) IBP in U251MG cells. Data are presented as mean + SEM, *n* = 3–4. A two-way ANOVA with a Bonferroni post-test was performed. Differences were considered significant at *p* < 0.05. * = *p* < 0.05; ** = *p* < 0.01; *** = *p* < 0.001.

Previous studies have shown that glioma cells are responsive to exogenous prostaglandins including PGE_1_, PGE_2_, PGD_1_, and PGD_2_ [8,34]. In a targeted lipidomic study we found PGE_2_ to be the major prostaglandin produced by human GBM tissue [13]. In Figure 2 the effects of PGE_2_ on cell counts are shown for three GBM cell lines, U87MG, U251MG and T98G. The presence of exogenous PGE_2_ significantly increased the total number of viable U87MG cells after 48 h at 10 μM and after 72 h at both 1 μM and 10 μM (Figure 2A). U251MG cell counts increased after 48 h and after 72 h at both 1 μM and 10 μM (Figure 2B). T98G cell counts increased after 48 h and 72 h at 10 μM (Figure 2C).

### 2.3. Effect of the Non-Specific Cyclooxygenase Inhibitor, Ibuprofen, on GBM Cell Counts

The effects of the non-specific cyclooxygenase inhibitor, ibuprofen (IBP), on glioma cell counts are presented in Figure 2 for the GBM cell lines U87MG and U251MG. Previous studies have shown that IBP has significant inhibitory effects on cell counts in T98G cells. In addition, IBP caused reduced mitotic rates, reduced BrdU incorporation and increased apoptotic rates in T98G cells [8]. IBP caused a significant reduction in cell counts after 24 h at 100 μM (Figure 2D,E) for both U87MG and U251MG cells. After 48 h and 72 h, IBP caused a significant, dose-dependent reduction in cell counts at all concentrations tested from 25–100 μM for both U87MG and U251MG cells (Figure 2D,E).

### 2.4. Effect of the Specific Cyclooxygenase Inhibitors, SC560 (COX1) and NS398 (COX2), on GBM Cell Counts

**Figure 3:**
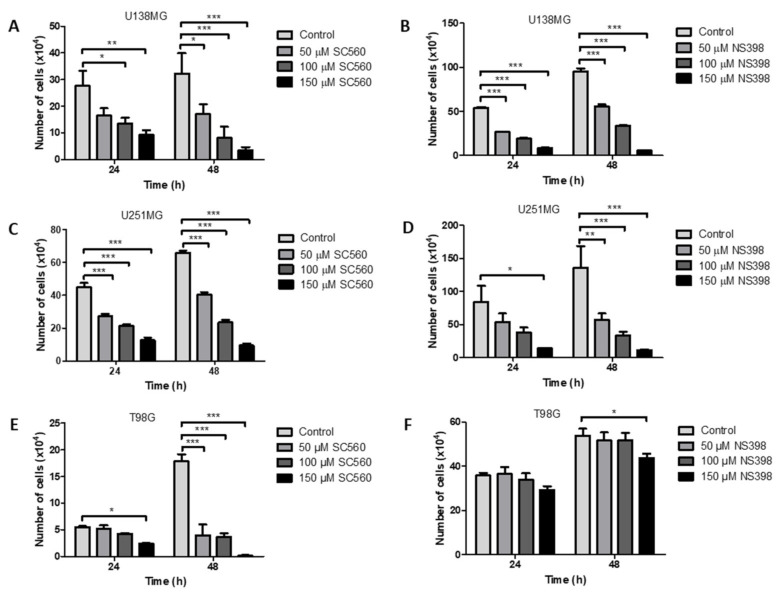
Effects of COX1 inhibitor, SC560, and COX2 inhibitor, NS398, on GBM cells counts. Graphs show the results after 24 or 48 h of treatment with SC560 or NS398. (**A**) SC560 in U138MG cells; (**B**) NS398 in U138MG cells; (**C**) SC560 in U251MG cells; (**D**) NS398 in U251MG cells; (**E**) SC560 in T98G cells; (**F**) NS398 in T98G cells. Data are presented as mean + SEM, *n* = 4–8. A two-way ANOVA with a Bonferroni post-test was performed. Differences were considered significant at *p* < 0.05. * = *p* < 0.05; ** = *p* < 0.01; *** = *p* < 0.001.

The effects of the COX1 inhibitor SC560 on glioma cell counts are presented in Figure 3 for the GBM cell lines U138MG, U251MG and T98G. In U138MG cells SC560 caused a significant dose-dependent inhibition of cell counts at both 24 h and 48 h (Figure 3A). A similar result was seen for U251MG cells (Figure 3C). T98G cells were less sensitive to SC560 at 24 h than the other two cell lines but were significantly dose-dependently inhibited after 48 h (Figure 3E). In the case of the specific COX2 inhibitor NS398, a significant dose-dependent inhibition of cell counts was seen at 24 h and 48 h for U138MG (Figure 3B). NS398 caused inhibition in U251MG after 24 h only at 150 μM and caused a significant dose-dependent inhibition at all concentrations after 48 h (Figure 3D). T98G cells did not show significant changes in cell counts with NS398 at 24 h and at 48 h only 150 μM caused inhibition of cell counts (Figure 3F).

### 2.5. Cyclooxygenase Inhibition Alters Cell Cycle in GBM Cell Lines

**Figure 4:**
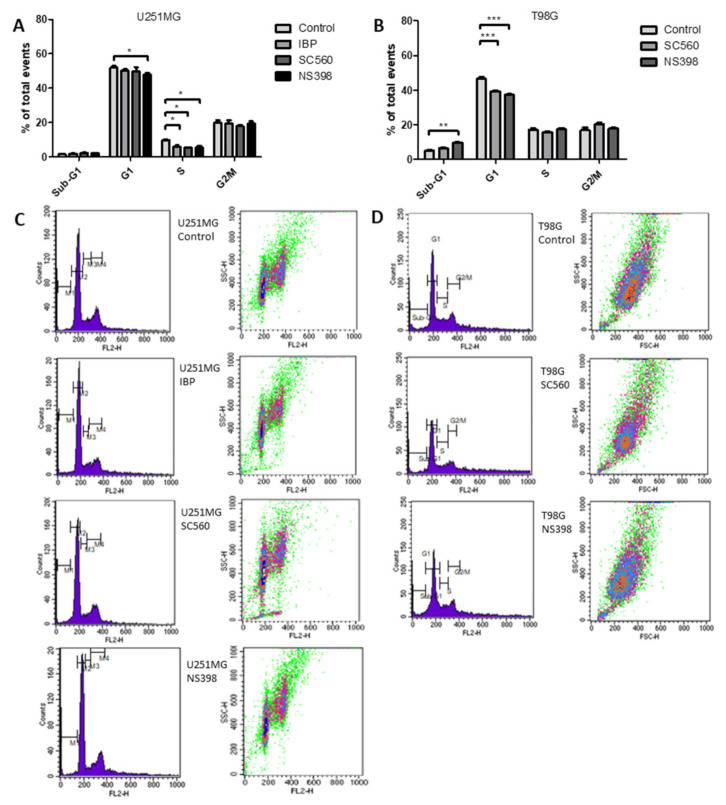
COX inhibitors alter cell cycle distribution. Cells were treated with IBP (50 μM), SC560 (50 μM) or NS398 (50 μM) for 48 h before propidium iodide staining and flow cytometer analysis. (**A**) Cell cycle distribution in U251MG cells; (**B**) cell cycle distribution in T98G cells; (**C**) Flow cytometry dot plots for U251MG control, IBP, SC560 and NS398 treatments; (**D**) Flow cytometry dot plots for T98G control, SC560 and NS398 treatments. Data are presented as mean + SEM, *n* = 3. A two-way ANOVA with a Bonferroni post-test was performed. Differences were considered significant at *p* < 0.05. * = *p* < 0.05; ** = *p* < 0.01; *** = *p* < 0.001.

To compliment the data concerning cell counts, cell cycle analysis was performed using propidium iodide staining and detection by flow cytometry. In U251MG cells IBP caused a significant reduction in S-phase. A similar reduction in S phase was found when U251MG cells were treated with SC560 or NS398. NS398 also caused a significant reduction in the G1 phase in U251MG cells (Figure 4A,C). When T98G cells were treated with SC560 or NS398 there was a significant reduction in the G1 phase and a significant increase in the sub-G1 phase was found with NS398 treatment (Figure 4B,D).

### 2.6. Cyclooxygenase Inhibition Alters Cell Migration in GBM Cell Lines

**Figure 5:**
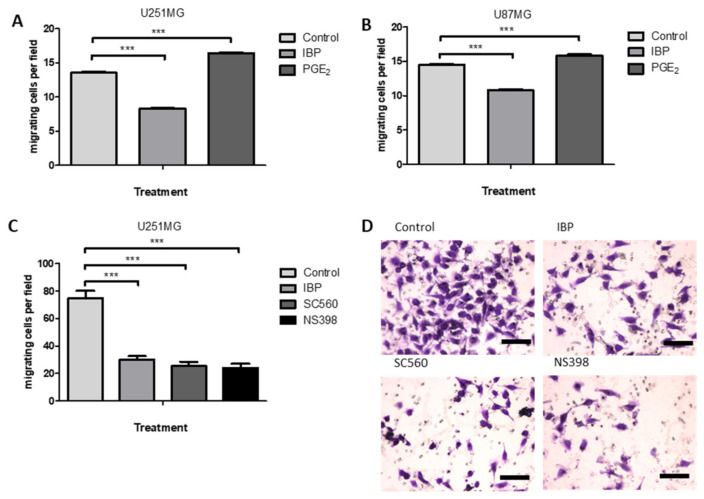
Opposing effects of PGE_2_ and COX inhibitors on GBM cell migration. Cells were treated with PGE_2_ (10 μM), IBP (25 μM), SC560 (50 μM) or NS398 (50 μM) for 48hours, with the final 12 h in a transwell assay. (**A**)PGE_2_ and IBP in U251MG cells; (**B**) PGE_2_ and IBP in U87MG cells; Cells were treated with IBP (50 μM), SC560 (50 μM) or NS398 (50 μM) for 48hours, with the final 12 h in a transwell assay. (**C**) IBP, SC560 and NS398 in U251MG cells using double the cell density in (**A**). (**D**) Images of transwells for U251MG control, IBP, SC560 and NS398 treatments. A two-way ANOVA with a Bonferroni post-test was performed. Data are presented as mean + SEM, *n* = 4–8. Differences were considered significant at *p* < 0.05; *** = *p* < 0.001. Scale bar in D = 30 µm.

Previous studies have shown that both PGE_1_ and PGE_2_ can stimulate the migration of T98G GBM cells [8] and that PGD_2_ can stimulate the migration of U251MG and U87MG cells [34]. In Figure 5, the effects of COX inhibition on cell migration in a transwell assay are shown for U87MG and U251MG, with PGE_2_ stimulation as a positive control. In Figure 5A,B IBP caused a significant inhibition of cell migration in U251MG and U87MG cells, respectively. As expected, exogenous PGE_2_ caused an increase in cell migration. The presence of SC560 or NS398 had an even greater inhibitory effect on cell migration in U251MG cells in comparison with the non-specific inhibitor IBP (Figure 5C).

### 2.7. Inhibition of Prostaglandin E_2_ Receptors EP2 and EP4 Decrease Cell Counts in GBM Cell Lines

**Figure 6:**
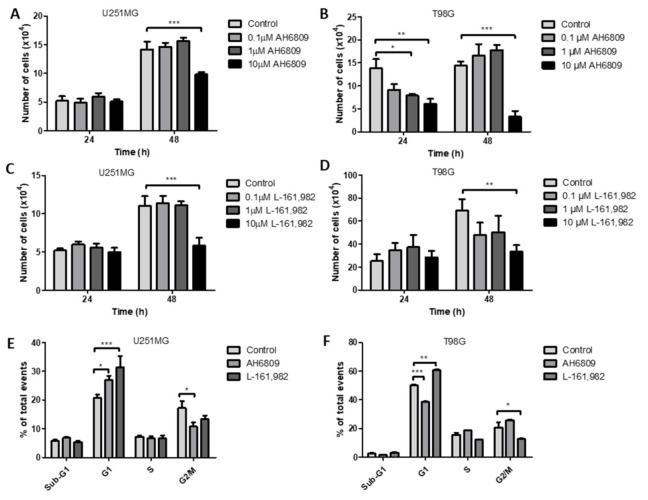
Effects of PGE_2_ receptor EP2 or EP4 receptor antagonists, AH6809 and L-161,982, on cell counts and cell cycle distribution. Graphs show the results after 24 or 48 h of treatment with AH6809 or L-161,982 in A–D. (**A**) AH6809 in U251MG cells; (**B**) AH6809 in T98G cells; (**C**) L-161,982 in U251MG cells; (**D**) L-161,982 in T98G cells; Cells were treated with AH6809 (10 μM) or L-161,982 (10 μM) for 48 h before propidium iodide staining and flow cytometer analysis in E and F; (**E**) Cell cycle distribution in U251MG cells; (**F**) Cell cycle distribution in T98G cells. Data are presented as mean + SEM, *n* = 3. A two-way ANOVA with a Bonferroni post-test was performed. Differences were considered significant at *p* < 0.05. * = *p* < 0.05; ** = *p* < 0.01; *** = *p* < 0.001.

The prostaglandin receptor antagonist AH6809 has almost equal affinity for the EP1, EP2, EP3-III and DP1 receptors while L-161,982 is a high affinity EP4 antagonist [35,36]. In the GBM cell lines used throughout the study the predominantly expressed prostaglandin E series receptors are the EP2 and EP4 receptors. Previous studies have also shown that while the prostaglandin D series DP2 receptor is present, the DP1 receptor is only present in U251MG and U87MG [34]. Thus, in our study, AH6809 is effectively an inhibitor of the prostaglandin E series EP2 receptor present in the cells under investigation, since neither EP1 nor EP3-III are present. In U251MG cells AH6809 caused a significant decrease in cell counts after 48 h at 10 μM (Figure 6A). In T98G cells AH6809 caused a significant reduction in cell counts after 24 h and 48 h at 10 μM (Figure 6B). The EP4 receptor antagonist L-161,982 caused a significant inhibition of cell counts after 48 h at 10 μM in both U251MG and T98G cells (Figure 6C,D).

### 2.8. Inhibition of EP2 and EP4 Receptors Alters Cell Cycle in GBM Cell Lines

The EP2 receptor antagonist AH6809 caused a significant increase in the G1 phase and a significant decrease in the G2/M phase in U251MG cells. The EP4 antagonist L-161,982 caused a significant increase in the G1 phase in U251MG cells (Figure 6E). In T98G cells AH6809 caused a significant decrease in the G1 phase, while L-161,982 caused a significant increase in the G1 phase and a significant decrease in the G2/M phase (Figure 6F).

### 2.9. Effects of EP Receptor Inhibition on Cell Migration in GBM Cell Lines

**Figure 7:**
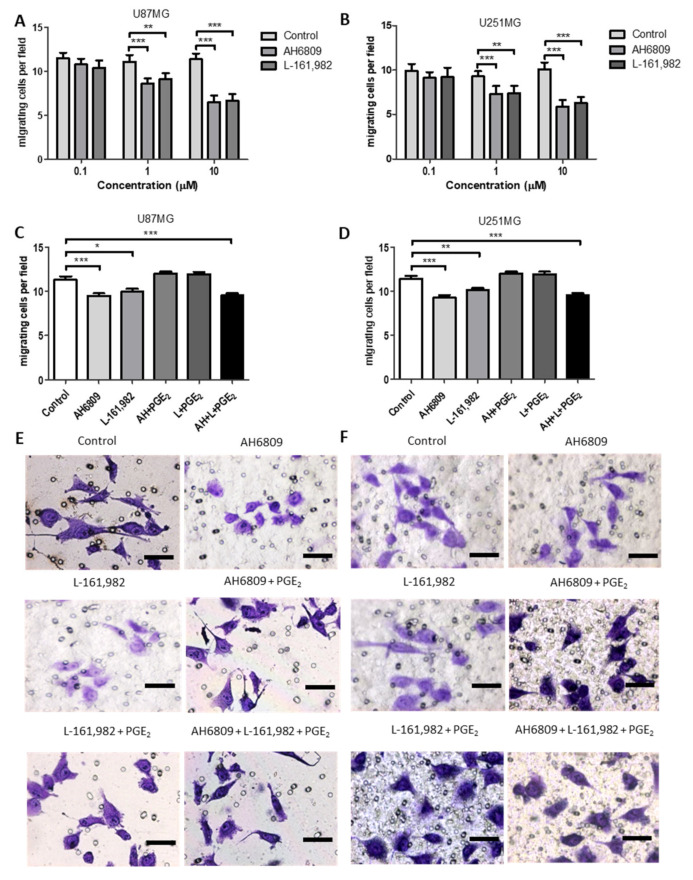
Effects of EP2 or EP4 receptor antagonists, AH6809 and L-161,982, on GBM cell migration in the presence or absence of exogenous PGE_2_. Graphs show the results of EP receptor antagonism in the absence or presence of exogenous PGE_2_. Cells were treated for 12 h with EP receptor antagonists in (**A**,**B**). (**A**) Concentration dependent inhibition of cell migration by EP2 or EP4 antagonists in U87MG; (**B**) Concentration dependent inhibition of cell migration by EP2 or EP4 antagonists in U251MG; Cells were treated for 1 h with EP receptor antagonists before addition of PGE_2_ for an additional 11 h, totalizing 12 h treatment with antagonists in C-F. (**C**) EP receptor antagonists in the absence or presence of exogenous PGE_2_ in U87MG cells; (**D**) EP receptor antagonists in the absence or presence of exogenous PGE_2_ in U251MG cells; (**E**) Images of transwells for U251MG control, AH6809 (1 μM), L-161,982 (1 μM), AH6809 (1 μM) + PGE_2_ (5 μM), L-161,982 (1 μM) + PGE_2_ (5 μM) and AH6809 (1 μM) + L-161,982 (1 μM) + PGE_2_ (5 μM) treatments; (**F**) Images of transwells for U251MG control, AH6809, L-161,982, AH6809 + PGE_2_, L-161,982 + PGE_2_ and AH6809 + L-161,982 + PGE_2_ treatments. Data are presented as mean + SEM, *n* = 3, in triplicate. A two-way ANOVA with a Bonferroni post-test was performed. Differences were considered significant at *p* < 0.05. * = *p* < 0.05; ** = *p* < 0.01; *** = *p* < 0.001. Scale bar in E and F = 30 µm.

In U87MG and U251MG cells AH6809 and L-161,982 caused a significant concentration dependent decrease in cell migration in the transwell assay (Figure 7A,B). When the EP2 antagonist was used concomitantly with PGE_2_ the inhibitory effect was abolished in both U87MG and U251MG cells. A similar result was seen when the EP4 antagonist was used concomitantly with PGE_2_ in both cell lines (Figure 7C–F). These results suggested that when one EP receptor was blocked the other could still be stimulated by exogenous PGE_2_ to compensate for the inhibitory effects of the antagonist. This idea was tested by inhibiting the EP2 and EP4 receptors concomitantly with PGE_2_. In this case the inhibitory effect of EP2 and EP4 antagonism on cell migration could not be reverted in the presence of exogenous PGE_2_ for U87MG or U251MG (Figure 7C–F).

### 2.10. MMP2 and MMP14 Expression in GBM

Considering the significant effects of PGE_2_ on cell migration and the importance of cyclooxygenase inhibitors in the control of cell growth, cell cycle and migration shown above we next chose to explore an area which is poorly understood in GBM, which is the control of matrix metalloproteinase expression and activity by the prostanoid pathway.

**Figure 8:**
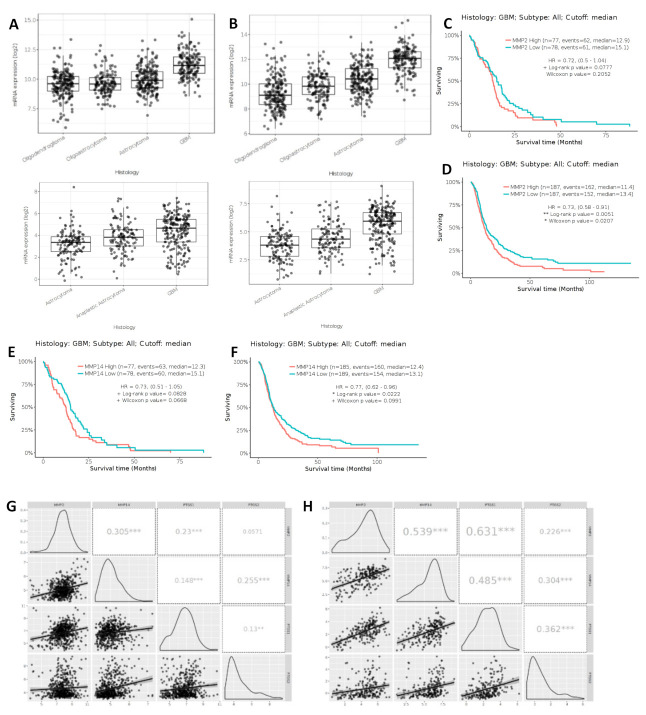
Matrix metalloproteinase (MMP) expression in gliomas. (**A**) MMP2 mRNA expression in gliomas using Gliovis software for analysis, TCGA and CCGA datasets; (**B**) MMP14 mRNA expression in gliomas using Gliovis software for analysis, TCGA and CCGA datasets. Kaplan-Meier survival curves for GBM tumors, TCGA and CCGA datasets. (**C**) MMP2 expression, RNASeq TCGA; (**D**) MMP2 expression, CCGA; (**E**) MMP14 expression, RNASeq TCGA; (**F**) MMP14 expression, CCGA; (**G**) Correlation between MMP2, MMP14, PTGS1 and PTGS2 in GBM, TCGA; (**H**) Correlation between MMP2, MMP14, PTGS1 and PTGS2 in GBM, CCGA. For Kaplan-Meier curves high and low expression was based on median expression levels for each protein. The log-rank Mantel-Cox test was used with differences considered significant at *p* < 0.05. For correlation curves-Pearson correlation coefficient with significance: *** *p* < 0.001, ** *p* < 0.01.f.

The mRNA expression and RNASeq of matrix metalloproteinase 2 (MMP2) and matrix metalloproteinase 14 (MMP14) is presented for differing grades of glioma using the Gliovis data portal for visualization and analysis of brain tumor expression datasets to analyze data from the TCGA and the CCGA [33]. The expression of both MMP2 and MMP14 are significantly increased in grade IV GBMs in comparison with lower grade gliomas (Figure 8A,B). The expression of MMP2 and MMP14 are significantly correlated with patient survival, where higher MMP expression is correlated with a poorer survival (Figure 8C–F). A comparison of the mRNA expression of COX1 and COX2 presented in Figure 1A,B was made with the expression of MMP2 and MMP14 presented in Figure 8 using data from both the TCGA and the CCGA (Figure 8G,H). This analysis identified a significant positive correlation between the expression of MMP2 and MMP14 in GBM tissue samples. This correlation could be expected considering the biological relevance of MMP14 activity to the activation of MMP2. A significant positive correlation was also seen between MMP2 and PTGS1 (COX1) in both data sets, while a significant positive correlation was seen for MMP2 and PTGS2 (COX2) only in the CCGA data set. MMP14 was significantly positively correlated with both PTGS1 and PTGS2 in both data sets. Finally, PTGS1 and PTGS2 were also significantly positively correlated in both data sets.

### 2.11. MMP mRNA, Protein Expression and Activity

**Figure 9:**
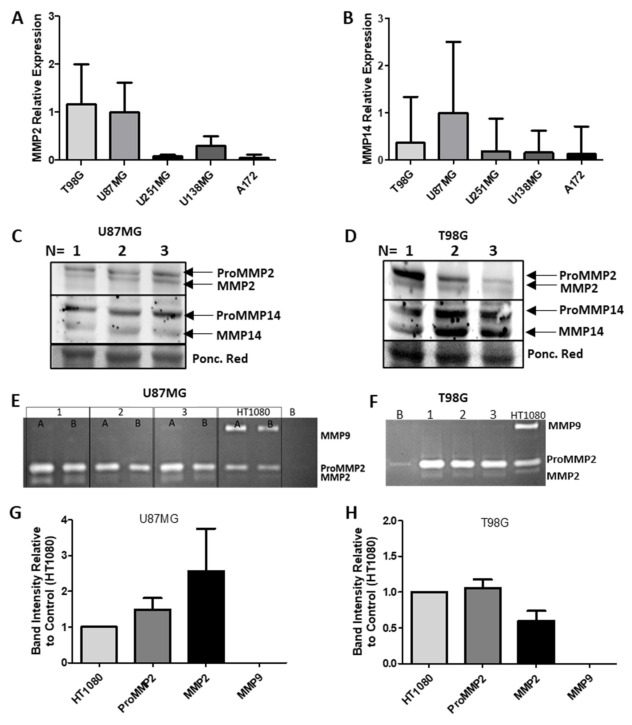
MMP expression in GBM cell lines. (**A**) MMP2 expression in T98G, U87MG, U138MG, U251MG and A172 GBM cell lines; (**B**) MMP14 expression in T98G, U87MG, U138MG, U251MG and A172 GBM cell lines; (**C**) Western blot of MMP2 and MMP14 protein expression in U87MG cells-proMMP2 (72 kDa), MMP2 (62 kDa), proMMP14 (63 KDa), MMP14 (60 KDa) normalized to Ponceau Red staining of the same membrane; (**D**) Western blot of MMP2 and MMP14 protein expression in T98G cells–proMMP2 (72 kDa), MMP2 (62 kDa), proMMP14 (63 KDa), MMP14 (60 KDa) normalized to Ponceau Red staining of the same membrane; (**E**) Gelatin zymography for MMP2 and MMP9 in U87MG; (**F**) Gelatin zymography for MMP2 and MMP9 in T98G; (**G**) Zymogram quantification of U87MG MMP activity relative to HT1080 positive control for MMP2 and MMP9 activity; (**H**) Zymogram quantification of T98G MMP activity relative to HT1080 positive control for MMP2 and MMP9 activity. Images in C and D are the DMSO controls presented in Figure 11. Data are presented as mean + SEM, *n* = 3. Part of these data have been published in [37].

Initially, we explored the mRNA expression of MMP2, MMP9 and MMP14 in the human glioma cell lines T98G, U87MG, U138MG, U251MG and A172 by real time RT-qPCR (Figure 9A,B). The two cell lines with the highest mRNA expression levels of MMP2 and MMP14 were selected to continue the study, these being U87MG and T98G. MMP9 mRNA was not amplified after 40 cycles by conventional RT-PCR and was undetectable in RT-qPCR [37]. Western blotting confirmed the presence of MMP2 and MMP14 protein in both cell lines (Figure 9C,D). Gelatin zymography demonstrated the presence of MMP2 activity and the absence of MMP9 activity in both U87MG and T98G (Figure 9E–H).

### 2.12. Influence of COX Inhibitors and PGE_2_ on MMPs

**Figure 10:**
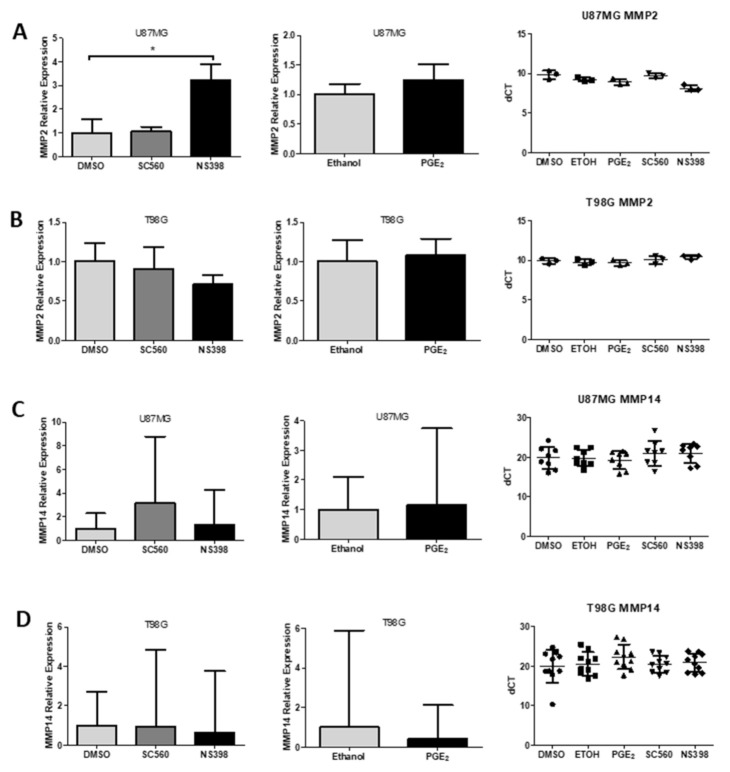
Effects of COX inhibitors on MMP2 and MMP14 mRNA expression in GBM cells. Cells were treated with SC560 (50 μM), NS398 (50 μM) or PGE_2_ (10 μM) for 24 h and mRNA expression was determined by RT-qPCR (**A**) MMP2 gene expression in U87-MG (**B**) MMP2 gene expression in T98G (**C**) MMP14 gene expression in U87-MG (**D**) MMP14 gene expression in T98G. Relative expression was calculated using 2^−ΔΔCT^ and dCT’s are also shown. Data are presented as mean + SEM, *n* = 3. One-way ANOVA or Student’s *t*-test were performed. Differences were considered significant at *p* < 0.05. * = *p* < 0.05.

After selecting the two cell lines with the highest MMP2 expression we examined the expression and activity of MMPs in the presence of specific COX inhibitors or exogenous PGE_2_. Specific COX inhibitors were used to test whether COX1 or COX2 inhibition could interfere with MMP2 or MMP14 expression. Real time quantitative PCR demonstrated that the COX1 inhibitor SC560 did not alter the expression of MMP2 and MMP14 mRNA in U87MG and T98G cells (Figure 10A–D). The COX2 inhibitor NS398 did not alter the expression of MMP2 or MMP14 in T98G cells (Figure 10C,D). However, NS398 produced an almost 3-fold increase of MMP2 mRNA in U87MG (Figure 10A). PGE_2_ did not alter the expression of MMP2 or MMP14 in U87MG or T98G cells (Figure 10A–D).

**Figure 11:**
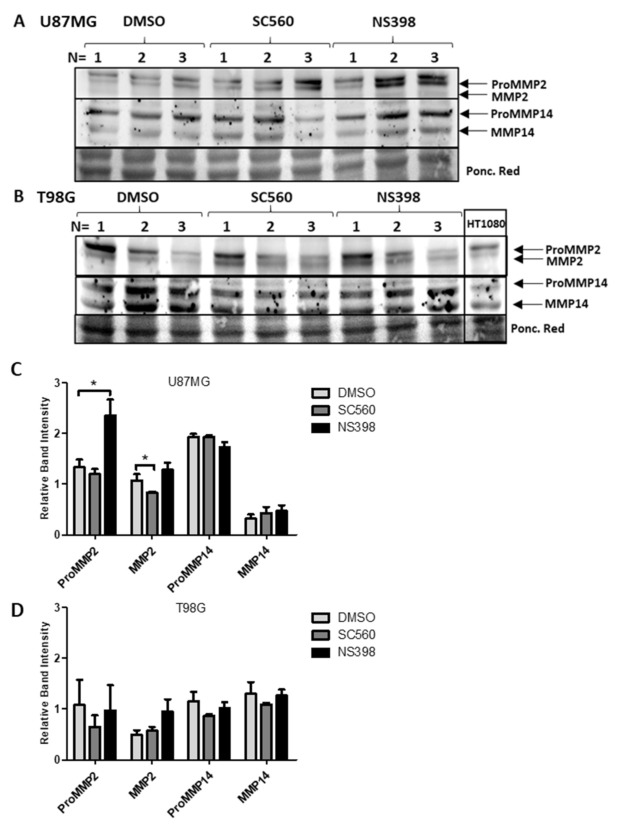
Western Blot and relative band intensities of GBM cells treated with COX inhibitors. Cells were cultivated with SC560 (50 μM) or NS398 (50 μM) for 24 h and total cell protein was extracted. Each lane represents a separately cultivated trial and contains 40 µg of protein. (**A**,**C**) U87MG cells; (**B**,**D**) T98G cells. (**C**,**D**) show the relative expression of proMMP2 (72 kDa), MMP2 (MMP-2: 62 kDa), proMMP14 (63 kDa) and MMP14 (60 kDa) normalized to Ponceau Red staining of the same membrane. Data are presented as mean + SEM, *n* = 3. A two-way ANOVA with a Bonferroni post-test was performed. Differences were considered significant at *p* < 0.05. * = *p* < 0.05.

**Figure 12:**
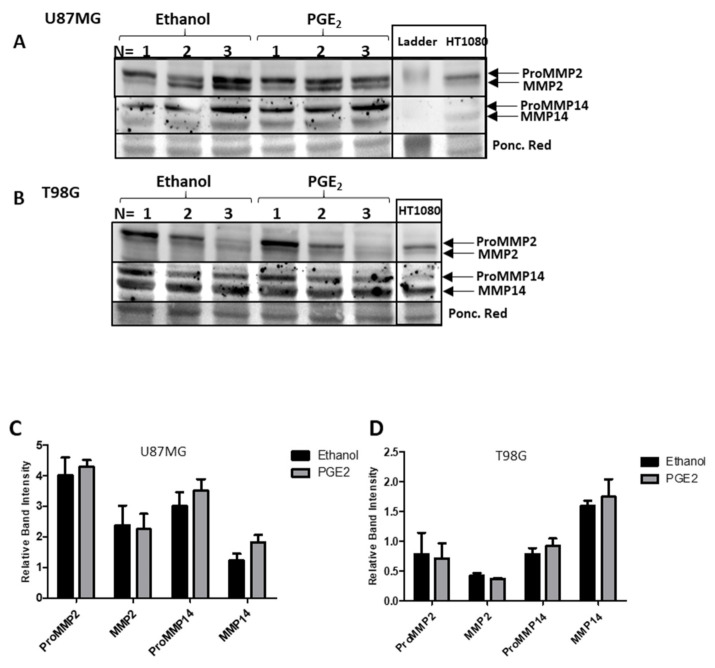
Western Blot and relative band intensities of GBM cells treated with exogenous PGE_2_. Cells were cultivated with PGE_2_ (10 μM) for 24 h and total cell protein was extracted. Each lane represents a separately cultivated trial and contains 40 µg of protein. (**A**,**C**) U87MG cells; (**B**,**D**) T98G cells. (**C**,**D**) show the relative expression of proMMP2 (72 kDa), MMP2 (MMP-2: 62 kDa), proMMP14 (63 kDa) and MMP14 (60 kDa) normalized to Ponceau Red staining of the same membrane. Data are presented as mean + SEM, *n* = 3. A two-way ANOVA with a Bonferroni post-test was performed. Differences were considered significant at *p* < 0.05.

**Figure 13:**
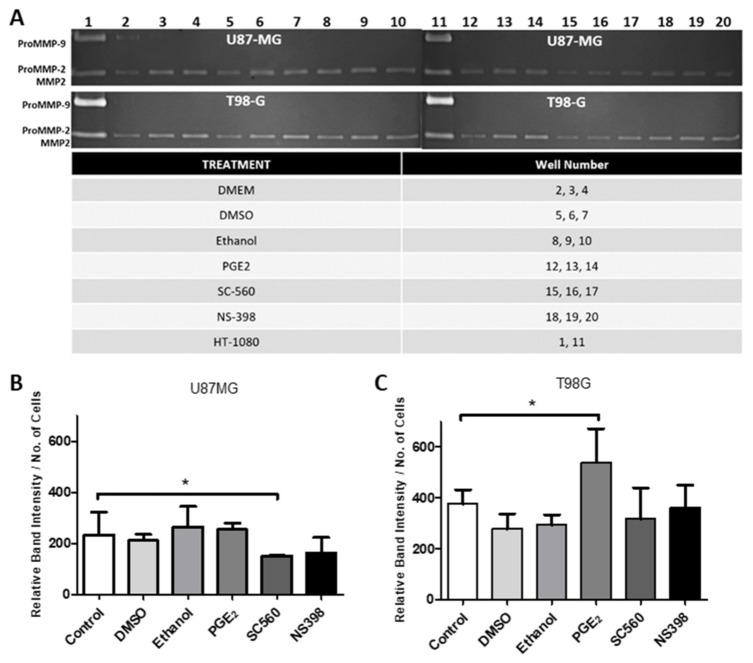
Gelatin zymography of GBM cells after treatment with exogenous PGE_2_ or COX inhibitors. (**A**) Cells were treated for 24 h with PGE_2_ (10 μM), SC560 (50 μM) or NS398 (50 μM) and the serum-free medium was used for gelatin zymography. Band intensity was normalized to the number of cells present after 24 h of treatment. (**B**) ProMMP2 activity in U87MG cells; (**C**) ProMMP2 activity in T98G cells. Data are presented as mean + SEM, *n* = 3. A two-way ANOVA with a Bonferroni post-test was performed. Differences were considered significant at *p* < 0.05. * = *p* < 0.05.

A significant decrease in MMP2 protein was identified by western blotting in U87MG cells after treatment with SC560 (Figure 11A,C). Western blotting further confirmed a significant increase in ProMMP2 protein caused by NS398 (Figure 11A,C). No alteration was seen in MMP14 protein expression in U87MG cells after treatment with SC560 or NS398 (Figure 11A,C). No influence of COX inhibitor treatments on MMP2 or MMP14 protein expression was observed in T98G (Figure 11B,D). PGE_2_ did not alter MMP2 or MMP14 protein expression in U87MG or T98G cells (Figure 12A–D). Gelatin zymography confirmed that SC560 significantly decreased latent MMP2 activity in U87MG cells (Figure 13A,B). In T98G cells PGE_2_ caused a significant increase in latent MMP2 activity (Figure 13A,C).

## 3. Discussion

The present study has confirmed the positive effects of PGE_2_ on cell proliferation and the inhibitory effects of the NSAID, IBP as previously seen for other GBM cell lines [8,21,22]. To distinguish the importance of COX2 relative to COX1 activity in GBM cell lines selective inhibitors were studied. The COX1 inhibitor SC560 caused a significant dose and time dependent inhibition of cell counts in GBM cells. A significant decrease in the S-phase of the cell cycle was identified in the presence of SC560 in U251MG cells, while a significant decrease in G1 was seen for T98G cells. The inhibition of COX1 also led to a significant inhibition of GBM cell migration in the transwell assay. The COX2 inhibitor NS398 caused a significant dose and time dependent inhibition of cell counts in GBM cells, although individual cell lines had a greater variability in their response than was seen for SC560. This variability may be due to the differences in COX2 expression among these cell lines as T98G typically expresses less COX2 than U87MG. NS398 also caused significant changes in the cell cycle, reducing G1 and S-phase in U251MG cells while increasing sub-G1 and decreasing G1-phase in T98G cells. The inhibition of COX2 by NS398 caused a significant inhibition of GBM cell migration in the transwell assay. These data prove that not only COX2, but also COX1 activity, is important to the normal function of GBM cells in in vitro conditions.

In GBM the main effects of PGE_2_ can be mediated through the EP1-EP4 receptors and the inhibition of these receptors may provide interesting targets for treatment. In the present study we identified the importance of both EP2 and EP4 receptors to the control of GBM cell proliferation and migration. The EP2 antagonist AH6809 caused a significant decrease in cell counts and a significant change in the cell cycle in GBM cells. EP2 antagonism also caused a significant concentration dependent inhibition of GBM cell migration. The EP4 receptor antagonist L-161,982 also caused a significant decrease in cell counts and a significant change in the cell cycle. EP4 receptor antagonism caused a significant concentration dependent inhibition of GBM cell migration. Interestingly, when one EP receptor was inhibited, the addition of exogenous PGE_2_ was sufficient to compensate the inhibition and return cell migration to control levels by stimulating the other EP receptor. However, when both EP2 and EP4 were inhibited by their respective antagonists concomitantly, the addition of exogenous PGE_2_ was unable to revert the inhibition of migration.

Considering the significant effects of PGE_2_ on cell migration and the importance of COX inhibitors in the control of cell growth, cell cycle and migration we then explored a poorly understood area of GBM cell biology, which is the control of matrix metalloproteinase expression and activity by the prostanoid pathway. In Chopra et al.’s review of MMPs in the CNS, they declare that MMPs function principally “in temporal modulation of inflammatory and immune processes by precise regulation of the bioactivity of signaling molecules and their pathways” [38]. Determining the relationship between MMP activity and cyclooxygenase inhibition is an important step in understanding the potential utility of the temporary and timely use of NSAIDs in GBM therapy.

When both MMP2 and MMP9 are synthesized, they are latent enzymes; 72 kDa (proMMP2) and 92 kDa (proMMP9). Latency is believed to be maintained by the presence of zinc at the catalytic domain bound to a sulfhydryl (SH) group present on a cysteine residue in the propeptide domain [25,39]. The disruption of this zinc-cysteine pairing, resulting from proteolytic intervention or conformational changes, results in the activation of these MMPs [40]. MMP14 (also known as MT1-MMP) is a membrane bound protein best known for cleaving proMMP2 into its active form, MMP2 [41].

The presence of MMP9 in immortalized GBM cell lines is a topic of debate, as observed in Hageman et al.’s review [29]. The data presented in this study demonstrate that MMP2 and MMP14 are clearly present within U87MG and T98G cells. These data coincide with the findings of other authors [29,42,43]. However, when examining Hagemann et al.’s table summarizing the authors who have reported the presence or absence of MMP9 in GBM cell lines, an obvious discrepancy between studies is observed [29]. According to the data presented in Figure 9 and Figure 13, and previous studies in our laboratory, MMP9 is not endogenously produced by either U87MG or T98G GBM cell lines [37]. All zymograms were run with HT1080 samples as a positive control for MMP expression [44]. Data in the study by Roomi et al. also did not find MMP9 in T98G cells [45]. One possible explanation for the differences between MMP9 expression in the cited studies could be the fact that cell culture conditions varied between studies (e.g., 2D, 3D, spheroid, in vivo, etc.), which could elicit different cellular responses of whether or not to produce MMP9 [46]. Despite the controversy and known limitations of in vitro GBM cell culture, MMP9 has been proposed as a potential cancer biomarker [47], and its high expression is associated with poorer survival rates in glioma patients [48].

Using the Gliovis portal [33] we identified correlations among COX1 and COX2 genes and MMP2, MMP9 and MMP14 genes in GBM tissues. This raised the question of whether COX inhibitors could interfere with MMP gene expression, protein expression and activity. For this we chose the two cell lines with the highest expression levels, U87MG and T98G. Hoping to observe the effects of COX inhibition on MMP expression and ECM-altering activity, U87MG and T98G cells were inhibited with SC560 (COX1 inhibitor) and NS398 (COX2 inhibitor) and stimulated with the major COX2 product, PGE_2_. After 24 h of treatment with NS398, qRT-PCR demonstrated an increase in MMP2 mRNA and western blots also showed a significant increase in ProMMP2 in U87MG (Figure 10 and Figure 11). PGE_2_ has been reported to upregulate the activity of MMP2 and endothelial tube formation in HUVEC cells, while COX2 inhibition by NS398 caused inhibition of tube formation [32]. Similarly, the inhibition of COX2 by celecoxib reduced MMP2 activity in an in vivo mouse model of endometriosis [32]. In the present study exogenous PGE_2_ increased the release of MMP2 into the extracellular environment of T98G cells (Figure 13).

MMP14 expression and synthesis were not influenced by COX inhibitors or exogenous PGE_2_ (Figure 10, Figure 11 and Figure 12). Interestingly, Kassem et al. reported that PGE_2_ reduces MMP14′s cleaving activity of ProMMP2 in fibroblasts through the EP4 receptor [49] This mechanism could explain why PGE2 treatments increased ProMMP2 and not the active MMP2 form (Figure 13). Data from our lab has demonstrated that EP4 is expressed in U87MG and T98G cells and the results obtained in the present study using antagonists to the EP2 and EP4 receptors show the importance of the EP4 receptor to the biology of the GBM cells studied (Figure 6 and Figure 7) [19]. Previous studies have demonstrated MMP14 can up-regulate COX2 expression through NF-κB in U87MG cells, despite MMP14′s catalytic function [50]. It should be noted that the effects seen on GBM cells with COX1 and COX2 inhibitors in vitro are obtained at concentrations above those required to inhibit the purified recombinant human enzymes, although these concentrations are similar to those used by many other studies of cancer cells in the literature.

COX1 is ubiquitously expressed in many tissue types, and functions as a homeostatic catalyst of bioactive lipid formation for cell-cell signalling and angiogenesis. The present study has demonstrated that COX1 inhibition influences MMP2 protein expression and ECM-altering activity in U87MG cells (Figure 11 and Figure 13). This is particularly interesting when compared with the correlation data presented in Figure 8, where MMP2 expression is significantly positively correlated with PTGS1 (COX1) in GBM tissue. This supports the hypothesis that a relationship may exist between COX1 and MMP2 in GBM cells which merits further investigation as a possible novel therapeutic target for drug development.

## 4. Materials and Methods

### 4.1. Cell Culture

The human glioma cell lines A172, U87MG, U138MG, U251MG, and T98G were analyzed at different points in the study. Previous studies have shown that all five cell lines express COX1 and COX2 [22]. For comparative analyses at certain points of this study, a human breast cancer cell line (MCF7), and a human fibrosarcoma cell line (HT1080) were used. All cell lines were cultured in DMEM (Dulbecco’s Modified Eagle Medium-Gibco, Thermo Fisher Scientific, Walsham, MA, USA) supplemented with 10% (*v/v*) FBS (fetal bovine serum–Gibco, Thermo Fisher Scientific, Walsham, MA, USA). The cells were maintained at 37 °C in a humidified atmosphere with 5% CO_2_ in both 25 cm^2^ and 75 cm^2^ flasks, until the desired confluency. Cultivation was performed following a previously established protocol [34,37].

### 4.2. Total RNA Extraction/cDNA Synthesis

For total RNA extraction and cDNA synthesis, the cell lines were processed following our previously established protocol [37]. The purified RNA was stored in a −80 °C freezer. Complementary DNA (20 μL) was obtained by using Moloney Murine Leukemia Virus Reverse Transcriptase (M-MLV RT) in a reverse transcription polymerase chain reaction (RT-PCR) with 2 μg of the RNA of interest. This reaction included 2 μL of a free nucleic acid mix (dNTP mix), 2 μL Random Primer, 1 μL of RNA inhibitor (RNAse OUT), 2 μL Dithiothreitol (DTT), 4 μL RT buffer, and 1 μL MMLV (all reagents were purchased from Invitrogen, Thermo Fisher Scientific, Walsham, MA, USA). An Eppendorf MasterCycler^®^ thermocycler was used to amplify the cDNA. Amplification was then confirmed by electrophoresis with a 1% agarose gel containing ethidium bromide revealed in a Syngene G-Box^®^ (UV light) and captured by the GeneSys program (Syngene, Daly City, CA, USA).

### 4.3. RT-PCR and RT-qPCR

To analyze the gene expression of the enzymes, Real Time quantitative PCR (RT-qPCR) and conventional RT-PCR were used. Primers were designed using the open-source Perlprimer program [51] and the NCBI Primer-BLAST tools. The primers for MMP-2, MMP-9, and MMP-14 were purchased from Invitrogen, Thermo Fisher Scientific, MA, USA and were initially tested using conventional RT-PCR and then confirmed by RT-qPCR. The real-time reactions were prepared containing Syber Green Mix (Applied Biosystems, Thermo Fisher Scientific, Walsham, MA, USA). The amplifications were performed using the 7300 REAL TIME PCR system (Applied Biosystems, Thermo Fisher Scientific, Walsham, MA, USA). Dissociation curves verifying amplification specificity were also performed. To evaluate the differential expression of the treated groups, the relative quantification method with 18s was used an endogenous control. The details of this protocol have already been established in a previous publication [34,37]. Primer sequences used were as follows:-

MMP2 forward, GAC CAG AAT ACC ATC GAG ACC A; MMP2 reverse, GTG TAG CCA ATG ATC CTG TAT GTG; 128 bp

MMP 9 forward, TTT GTT CAA GGA TGG GAA GTA CTG; MMP 9 reverse, CTC CTC AAA GAC CGA GTC CA; 124 bp

MMP 14 forward, CTT CAA AGG AGA CAA GCA TTG G; MMP 14 reverse, CCC TTG TAG AAG TAA GTG AAG AC; 297 bp

### 4.4. Cell Counts Assay

Cells were seeded at 3–4 × 10^4^ in a 24-well plate and after 24 h the cells were treated with SC560 (50 µM), NS398 (50 µM), AH6809 (10 µM) or L-161,982 (10 µM) (Cayman Chemical, Ann Arbor, MI, USA). The reagents were freshly prepared in DMSO for each experiment and DMSO was used as the vehicle control (Sigma Aldrich, São Paulo, Brazil). The cells and medium were collected at 24 h, 48 h, and 72 h and stained with 0.4% trypan blue to distinguish viable from unviable cells (Sigma Aldrich, São Paulo, Brazil). Cell counts were performed in a Neubauer chamber. All treatments were tested at least three times in triplicate.

### 4.5. Cell Cycle Assay—Propidium Iodide Fluorescence

Cells were seeded at 5 × 10^4^ for U251-MG and T98G in a 24-well plate. After 24 h the cells were treated with SC560 (50 µM), NS398 (50 µM), AH6809 (10 µM) or L-161,982 (10 µM) (Cayman Chemical, Ann Arbor, MI, USA). After 48 h of treatment the cells were trypsinized, followed by centrifugation at 1500 rpm for 5 min. The cell pellet was rinsed with ice-cold PBS, centrifuged and resuspended in ice-cold 70% ethanol for at least 24 h. After ethanol fixation, cells were again washed with ice-cold PBS and incubated for 30 min with 500 μL of a staining solution (20 μg/μL propidium iodide, 50 μg/μL RNAse A and 0.1% Triton X-100). At the end of incubation, cells were centrifuged, resuspended in ice-cold PBS and kept on ice before analysis. Cell cycle phase was determined by propidium iodide fluorescence detection of 10,000 events in a Becton Dickinson FACScalibur flow cytometer (Becton Dickinson, Franklin Lakes, NJ, USA) using 530 nm as the excitation wavelength (λ_ext._).

### 4.6. Transwell Migration Assay

The transwell migration assay was performed using a 24-well plate with 8.0 μm pore polycarbonate membrane Transwell inserts (Corning, Corning, NY, USA). Initially, U87MG or U251MG cells were seeded onto the top compartment at 2 or 3 × 10^4^ cells/well. Cells were kept at 37 °C in a humidified atmosphere with 5% CO_2_ for 6 h until treatment with IBP (25–50 µM), SC560 (50 µM), NS398 (50 µM), AH6809 (10 µM), L-161,982 (10 µM), or PGE_2_ (5–10 µM) (Cayman Chemical, Ann Arbor, MI, USA). After 12 h of treatment, the inserts were washed with PBS, then cells in the upper compartment were physically detached with a cotton swab. The inserts with remaining cells in the bottom compartment were stained with crystal violet in 10% ethanol. Polycarbonate membranes were mounted on glass slides with Entellan (Merck/Millipore), and images of 10 different random fields per membrane were captured with a coolSNAP-Pro camera (Media Cybernetics, Rockville, MD, USA) attached to a Nikon Optiphot-II microscope.

### 4.7. Protein Sample Preparation

U87MG and T98G cells were cultivated in 25 cm^2^ plates until they reached 80–90% confluency. At this point, cells were treated with PGE_2_ (10 µM), COX-1 Inhibitor SC560 (50 µM) or COX-2 Inhibitor NS398 (50 µM) with their respective vehicle controls, in duplicates, for 24 h following the methodology previously described. Cells were then trypsinized and counted in a Neubauer chamber. Next, a pellet was formed by centrifugation at 4 °C for 3 min at 410 RCF and washed with cold PBS 3x before snap-freezing the samples in liquid nitrogen and stored at −80 °C until the time of extraction.

The frozen pellet was slowly thawed on ice then lysing buffer containing a protease inhibitor mix (cOmplete protease inhibitor, Roche, Indianapolis, IN, USA) was added and the pellet was re-suspended for 5 min and left on ice for 30 min. After centrifugation at 9520 RCF in 4 °C for 10 min, the absorbance of the samples was read at 750 nm, the protein content of the sample of interest was determined against a protein concentration curve of bovine serum albumin. Finally, 4× Laemmli sample buffer was added (1:1) and the samples were heated (95 °C) for 5 min. Then the samples of total protein extract were frozen at −80 °C for long-term storage.

### 4.8. Western Blot

40 μg of protein from each sample were loaded onto 10% SDS-PAGE gels and after electrophoretic separation were transferred to nitrocellulose membranes. The transfer was confirmed by staining the membrane with 0.5% Ponceau red solution. Immunoblotting followed standard methods which were briefly as follows (B). The washed membranes were blocked with 5% (*w/v*) fat free milk for 1hr and after removal of excess blocking solution by washing, the membranes were incubated overnight with the primary antibodies (MMP-2 (1:2000) R&D Systems, USA; MMP-14 (1:2000) R&D Systems, USA; actin (1:3000) Abcam, Cambridge, MA, USA). The membranes were washed and incubated with secondary antibodies for 2 h at room temperature (anti-goat-HRP (MMP-2), anti-mouse-HRP (MMP-14), anti-rabbit-HRP (actin) (1:2000)). Finally, the membranes were and developed with enhanced chemiluminescence (ECL- Bio-Rad Laboratories Inc., São Paulo, Brazil) in a Syngene G-BOX with its GeneSys program. The ImageJ program was used to determine relative band intensities.

### 4.9. Zymogram Assay

Gelatin zymography assays measure degradation caused by gelatinase enzymes, such as Matrix Metalloproteinases (MMPs) 2 and 9. To identify if MMPs 2 and 9 were produced by the cells, a zymogram was performed of the serum-free medium in which the cells were cultivated. The 10% SDS polyacrylamide gel (containing 1% Gelatin-Synth, São Paulo, Brazil) was prepared as recommended by Toth and Fridman [44]. Fresh serum-free medium samples were removed from cell culture after 21 h of incubation and placed on ice. The samples were then centrifuged at 10,000 RCF for 5 min at 4 °C. The supernatant was separated and used for the zymogram.

The samples were the diluted 1:1 with 2× Sample (Laemmli) Buffer, not containing Dithiothreitol (DTT)/mercaptoethanol, and were not boiled; all of which may denature the MMPs structure and subsequent enzyme activity beyond recovery. After resting for 5 min, 25 µL of the sample were placed in the well of the loading gel and ran for 2.5 h at 100 V. The gels were washed with 2.5% Triton X-100 Buffer three times for 15 min. The gels were then incubated for 17 h in development buffer (1.5M Tris-HCl, pH8.8 + 1M CaCl_2_ + 2% NaN_3_ + ddH_2_O) at 37 °C. The negative control was an identical PAGE performed at the same time, washed with 10 mM ethylenediaminetetraacetic acid (EDTA)-containing 2.5% Triton X-100 Buffer and incubated with 10 mM EDTA-containing development buffer, which is a potent inhibitor of MMP enzyme activity due to calcium and zinc chelation [52].

After incubation, the gels were washed with a fixation buffer (4.5:1:4.5 ratio of methanol, acetic acid, ddH_2_O) and stained using 0.1% Coomassie Blue staining buffer (4.5:1:4.5 ratio of methanol, acetic acid, ddH_2_O + Coomassie Blue) for 30 min. Then, the gels were washed in destaining buffer, equal to the fixation buffer, until bands could be seen. Images were captured in a Syngene G:BOX-GeneSys program. The ImageJ program (Version 1.52P. NIH, Bethesda, MD, USA) was used to determine relative band intensities.

### 4.10. Immunohistochemical (IHC) Analysis by Light Microscopy

Five-micrometer sections were cut from tumours in paraffin blocks on a microtome (Leica RM2145) and mounted on gelatinized slides. Several sections were used for many of the patients as multiple blocks were available for analysis. The slides were deparaffinized and rehydrated by standard xylene/alcohol/H_2_O procedures and the endogenous peroxidase was blocked with 3% methanolic H_2_O_2_ [15]. Non-specific binding sites were blocked with a solution of 2% bovine serum albumin/2% pre-immune donkey serum/PBST (PBS 0.1M, triton X-100 0.2%). The sections were incubated at room temperature overnight with the respective primary antibody produced in rabbit (COX1-1:250, COX2-1:250) (Abcam, USA) diluted in PBST. The negative controls received only PBST. The slides were washed with PBST and were incubated with anti-rabbit secondary antibodies produced in donkey (1:1000) (Abcam, USA) for 90 min. The slides were washed again with PBST and incubated with streptavidin-HRP (1:100) (GE-Amersham Biosciences, Amersham, UK) for 60 min. The reactions were developed with 3,3′-diaminobenzidine (DAB) (0.04%) plus H_2_O_2_ (0.03%). All slides were counterstained with haematoxylin, dehydrated and mounted with Permount (Fisher Scientific, Walsham, MA, USA). Images were captured with a coolSNAP-Pro camera (Media Cybernetics, Rockville, MD, USA) attached to a Nikon Optiphot-II microscope (Nikon, Melville, NY, USA) and analyzed using ImageProPlus software (Rockville, MD, USA).

### 4.11. Immunocytochemical (ICC) Analysis by Light Microscopy

Cells were grown on glass coverslips until close to confluency, then fixed in 4% formaldehyde in 0.1M phosphate buffer saline. The coverslips were washed with H_2_O and the endogenous peroxidase was blocked with 3% methanolic H_2_O_2_. From this point on, the procedure was that previously described for the GBM tissue sections.

### 4.12. Patient Selection and Ethical Considerations

Patients with primary brain tumours were included in the study, which was approved by the Medical Ethics Commission of the Biomedical Institute of the University of Sao Paulo (947/CEP) and by the Medical Ethics Commission of the Santa Casa Hospital in Sao Paulo (162/10). Written informed consent was obtained from all patients. Gene expression and lipidomic data from these patient samples have been previously published [13].

### 4.13. Statistical Analysis

Real time PCR followed the Livak method of 2^−ΔΔCT^ [53]. GraphPad Prism 5.0 software (Version 5.0. Graph Pad Software, San Diego, CA, USA) was used to plot and analyze all data. Analysis between two groups was performed using Student’s *t*-test. Analysis between three or more groups used one-way ANOVA followed by Dunnett’s post-test. Analysis between three or more groups, considering two variables, were performed using two-way ANOVA followed by Bonferroni’s post-test. Differences were considered significant with *p* < 0.05. The significance of the *p*-value is represented in the figures by “*” (* < 0.05, ** < 0.01, *** < 0.001). For gene expression analysis of the TCGA and CCGA databases, the Gliovis software for analysis was used [33]. For Kaplan–Meier curves, high and low expression was based on median expression levels for each protein. The log-rank Mantel-Cox test was used with differences considered significant at *p* < 0.05. For correlation curves the Pearson correlation coefficient was used with significance: *** *p* < 0.001, ** *p* < 0.01, * *p* < 0.05.

## Data Availability

Data available on reasonable request.

## Abbreviations

AH6809	An EP and DP receptor antagonist
BrdU	Bromodeoxyuridine
CCGA	Chinese glioma genome atlas
CNS	Central nervous system
COX1	Cyclooxygenase 1
COX2	Cyclooxygenase 2
DP	Prostaglandin D series receptor
EP	Prostaglandin E series receptor
GBM	Glioblastoma
IBP	Ibuprofen
L-161,982	A selective EP4 antagonist
MMP2	Matrix metalloproteinase 2
MMP14	Matrix metalloproteinase 14
NS398	A selective COX2 inhibitor
NSAIDs	Non-steroidal anti-inflammatory drugs
PGD_1_	Prostaglandin D_1_
PGD_2_	Prostaglandin D_2_
PGE_1_	Prostaglandin E_1_
PGE_2_	Prostaglandin E_2_
PTGS1	Prostaglandin H synthase 1
PTGS2	Prostaglandin H synthase 2
RT-PCR	Reverse transcription-polymerase chain reaction
RT-qPCR	Real time quantitative polymerase chain reaction
SC560	A selective COX1 inhibitor
TCGA	The cancer genome atlas
